# The Role of Science in Advising the Decision Making Process: A Pathway for Building Effective Climate Change Mitigation Policies in Mexico at the Local Level

**DOI:** 10.3390/ijerph13050451

**Published:** 2016-04-27

**Authors:** Roberto Barraza, Gilberto Velazquez-Angulo, Edith Flores-Tavizón, Jaime Romero-González, José Ignacio Huertas-Cardozo

**Affiliations:** 1 Instituto de Ingeniería y Tecnología, Universidad Autónoma de Ciudad Juárez (UACJ), Ciudad Juárez, Chihuahua 32330, México; gvelazq@uacj.mx (G.V.-A.); edflores@uacj.mx (E.F.-T.); jromero@uacj.mx (J.R.-G.); 2 Centro de Investigación en Mecatrónica Automotriz (CIMA), Tecnológico de Monterrey Campus Toluca, Toluca, Estado de México 50110, México; jhuertas@itesm.mx

**Keywords:** urban climate change, climate change mitigation, science into policy, climate change mitigation policies

## Abstract

This study examines a pathway for building urban climate change mitigation policies by presenting a multi-dimensional and transdisciplinary approach in which technical, economic, environmental, social, and political dimensions interact. Now, more than ever, the gap between science and policymaking needs to be bridged; this will enable judicious choices to be made in regarding energy and climate change mitigation strategies, leading to positive social impacts, in particular for the populations at-risk at the local level. Through a case study in Juarez, Chihuahua, Mexico, we propose a multidimensional and transdisciplinary approach with the role of scientist as policy advisers to improve the role of science in decision-making on mitigation policies at the local level in Mexico.

## 1. Introduction 

The United States, China, Brazil, India, and various other countries have been unsuccessful at reducing their greenhouse gas emissions [1]. More than half of the global population lives in urban areas, and these areas are emerging as the first line of response in adapting to and mitigating climate change. Despite efforts toward carrying out risk assessments, establishing greenhouse gas emissions reduction targets, and providing policy frameworks oriented toward action, most cities have limited scientific information regarding the risk and potential for mitigating and adapting to climate change, which is considered one of the greatest challenges in human history. A science-based foundation such as that provided by the Intergovernmental Panel on Climate Change (IPCC) at the national level is also required at the city level [2,3]. 

In Mexico, a weak annual GDP growth rate and steep drops in oil production over the past ten years are driving the need to open the country’s energy sector to foreign and national investments, which could boost its gas and electricity sectors. Reforms in the power generation sector will also be critical for economic growth and environmental and social sustainability [4] 

Mexico is the 11th largest emitter of greenhouse gases (GHGs), accounting for approximately 2% of the world’s emissions, which amount to 683 million metric tons of carbon dioxide equivalent CO_2_e [5,6,7]. 

At the 2015 Paris Climate Change Summit Mexico committed to the ambitious target of reducing its GHG emissions by 22% by 2030 and 50% by 2050. To achieve these goals, two strategies have been identified in the electricity sector: the use of natural gas resources and renewable, and energy-efficiency technologies.

Urban areas account for more than half of global primary energy use and energy-related CO_2_ emissions. Considering direct and indirect emissions, urban areas account for 67%–76% of global energy use and 71%–76% of global energy-related CO_2_ emissions. As of 2011, more than 52% of the world’s population (roughly 3.6 billion) lived in urban areas. By 2050, the urban population is expected to increase to 5.6–7.1 billion, or 64%–69% of the world’s population. As a result of this growth, an urban land cover is projected to expand by 56%–310% between 2000 and 2030 [8,9]. This means that the majority of urban infrastructure has yet to be built, which presents challenges as well as opportunities regarding GHG mitigation, especially in developing countries. 

Urban areas are positioned to make key contributions toward aggressive national targets for emission reductions. This is possible because mayors, as decision makers, have the opportunity to exert a strong influence on the main policies that could lead to emissions reductions, such as building energy efficiency standards, urban planning, public transportation, and promotion of renewables to meet a portion of their municipality’s energy needs [10,11,12].

Decision makers frequently encounter complex issues, and the role of the scientists as policy advisers on these matters is not always clearly defined or is not part of the process at all [13]. In addition, some of the more mainstream issues, such as climate change mitigation, are still not resolved, and the characteristics of science and government are considered contradictory very often as shown in Table 1 [14]. In such complexity, scientists are regularly asked for advice.

Merton, in 1945 wrote on the role of scientists in policy making and addressed the lack of empirical data on the roles of experts on public policy. He suggested that common divergences between scientist and decision maker’s interaction were related to conflicts of values and the different modes in which bureaucratic and academic organization function [15,16].

Scientists’ roles as advisers have remained mostly theoretical. Existing theories about science systems can be used to study real policy advice process. Unfortunately, empirical proof for the roles and process described in those theories are very limited [17,18,19]. 

We present an empirical multidimensional and transdisciplinary approach of scientists as advisers on policy making such that climate change mitigation policies are intended to have positive technical, economic, environmental, and social impacts. The methodology presented was empirically derived trough a study case to highlight the importance of multidimensional and interdisciplinary approaches to propose and enact climate change mitigation policies supported by reliable science knowledge, under the assumption that a shared understanding of science and its implications would help resolve opposing points of view held by scientist and decision makers for the benefit of society. Researchers working as advisers to decision-makers can take advantage of the insights provided in this paper. 

## 2. Methodology

The study case presented in the manuscript is a combination of an exploratory and explanatory type and focused on each stage of the process of “scientist in the role of adviser” throughout the study case and present the outcomes of this research. The stakeholders (scientists, decision makers, government) involved in this process at the local level in Ciudad Juarez are very similar in their views towards science and government as shown in other studies [13,20], allowing us to present a methodology which in principle can be applied to other geographical areas in Mexico or internationally. 

Science-policy interfaces are avenues for finding solutions for environmental challenges throughout strengthening collaboration between research disciplines and public administrators [21]. Spruijt *et al.* [13] presented a literature overview of the interdisciplinary roles of scientific experts when advising policymakers on complex issues (such as air pollution, energy efficiency, public health) and in particular on the factor that influences these roles. 

We propose a multidimensional and interdisciplinary approach that consist of five dimensional (technical, economic, environmental, social, and political) methodological approach to building climate change mitigation policies, with an aim to provide reliable scientific information, based on the particular conditions of the urban areas, to the decision makers. The proposed methodology was derived by carrying out a case study on energy efficiency in Ciudad Juarez, Chihuahua, Mexico. 

The methodology presented was developed from a study case, which we consider having significant strengths such as novelty, testability, and empirical validity. These strengths arise from the intimate relationship with the empirical data and experience throughout the study case research. 

The independence from prior literature is particularly well-suited to this area of research in which most had been theoretical. Scientist roles as advisers have remained mostly theoretical. Unfortunately, empirical proof for the roles and process described in those theories are very limited [14,16]. The following subsection discusses the derivation of the proposed methodology while Section 3 addresses the application of the methodology regarding the case study.

### Multidimensional and Transdisciplinary Approach

Multidimensional methodologies for decision-making and for implementing environmental policies have been reported in the past [21]*.* Despite the efforts, these approaches have not been widely adopted for two main reasons: (1) their application is targeted at a particular problem and there is a lack of general application to different climate change adaptation or mitigation scenarios, and (2) there is a lack of successful cases reported in the literature at local governmental level [10,12,22,23,24].

**Figure 1:**
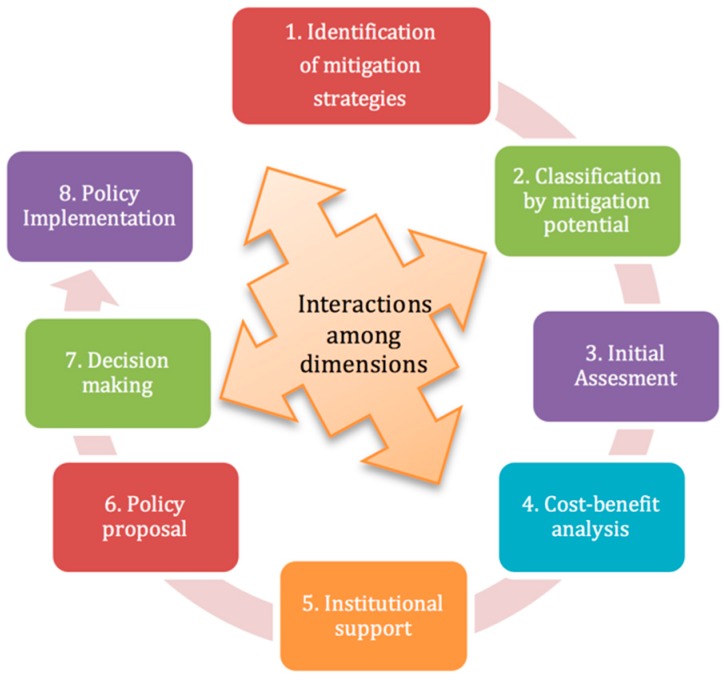
Proposed eight-step multidimensional methodology.

The proposed multidimensional methodology (Figure 1) consists of eight steps, each of which consists of interactions among technical, economical, social, political, and environmental dimensions, as shown in Table 2. It is important to highlight that these dimensions are related to each other in every step of the methodology.

In practice, the circular model shown is much more complex and iterative, and throughout the study case, the steps were not clearly separated as the model shows. The steps of the circular model were conceptualized retrospectively, trying to capture the whole process as best as possible in the eight steps presented in Figure 1. The dimensions considered on the proposed methodology are overlapping in several instances where more than one dimension influences most steps. We assumed that not one dimension was more important than the others. 

A description of the five dimensions involved in this methodology is as follows.

*1. Technical dimension:* The technical dimension refers to all quantitative and measured information derived from scientific knowledge, local indicators, technology roadmaps, journal publications, and case studies related to climate change mitigation and environmental policies in urban areas. It has become clear that climate change, which is one of the greatest challenges in human history, cannot be addressed by technical, scientific knowledge alone, although this knowledge is critical to understanding the physical phenomena involved. In this context, building relationships among different disciplines will improve the chances of success of public policies regarding climate change mitigation in urban areas [25].

*2. Environmental dimension:* The environmental dimension refers to the body of information about climate change mitigation potential and its indicators and impacts regarding the economic, social, and political dimensions. For this study, the environmental dimension is considered to be constrained by the characteristics of the particular urban areas for which public policies are being tailored.

*3. Economic dimension:* The economic dimension refers to the information derived from the financial analyzes of the proposed climate mitigation strategies and the opportunities and challenges involved in implementing the associated policy. Economics provides useful tools for assessing the benefits and costs of taking or not taking action for climate change mitigation, and of adaptation measures implemented to achieve competing societal goals. These understandings can help make policy decisions on climate change mitigation and can influence the actions taken by countries, institutions, and individuals [26]. Many economists believe that consumer choices reveal more about the economics of energy efficiency improvements than do engineering calculations. If engineering estimates of the potential energy savings from seemingly cost-effective investments leave out some costs or inappropriately model the consumer’s choices, then the assessment of the optimal approach from the consumer’s perspective will be incorrect. In that case, the engineering approach would overestimate the net benefits from energy efficiency investments [27].

*4. Social dimension:* The social dimension refers to the information derived from indicators that describe societal values and needs and the effects of these indicators on climate change mitigation. The significance of the social dimension and the role of ethics and economics are emphasized by Article 2 of the United Nations Framework Convention on Climate Change, which indicates that the ultimate objective of the convention is to avoid dangerous anthropogenic interference with the climate system [28]. IPCC considers that society is confronted with two main issues. The first is the question as to what constitutes ”dangerous interference” with the climate system, and the second one relates to how that interference should be dealt with [29]. Determining what is dangerous is not only a question for science; it also involves value judgments, which include ethics, economics, and other social sciences [26]. Ethics involve issues of justice and value. Justice is concerned with equity, fairness, and human rights. Value is a matter of worth, benefit, or good. Value can sometimes be quantified as, for example, a social welfare function or an index of human development. 

*5. Political dimension:* The political dimension refers to the political agenda prioritized according to pressures from the social, economic, and environmental dimensions, which influence decision makers’ policy choices.

## 3. Application of the Methodology to Our Case Study

Ciudad Juarez is the largest city in the state of Chihuahua, and the second most populated Mexican city on the US–Mexico border. Juarez has a population of approximately 1.5 million and is the eighth-largest metropolitan area in Mexico.

Approximately 40% of Chihuahua’s population lives in Juarez County, an area measuring approximately 4854 km^2^, which has the second-largest number of maquiladoras (factories that manufacture products for export) in Mexico. The growth of the maquiladora industry fueled an unprecedented increase in the local population, with job opportunities attracting people from Chihuahua and many other parts of the country [30,31]. 

In the framework of the proposed multidimensional methodology, we present a case study where the energy efficiency of street lighting was improved through the use of light-emitting diode (LED) lamps, with energy savings of 64% and emission reductions of ~30,000 tCO_2_e/year. These savings could be achieved by changing the 100,000 existing streetlights.

The successful application of our proposed methodology and promising results from the case study led us to the opportunity to work with the State Congress.

Step 1: Identification of Mitigation Strategies (Social, Political, and Environmental Dimensions)

This step involved identification of options for climate change mitigation strategies at the local governmental level (technical and environmental dimensions) by taking the social and political dimensions into account.

A marginal abatement cost curve (MACC) is a convenient way to present low-carbon options as alternatives to business-as-usual economic activities. An MACC can provide a brief overview of the potential and costs of low-carbon technologies for the economy as a whole or a particular sector [32].

Mexico’s MACC for the period of 2009–2030 [7,33] was used to identify approaches to climate change mitigation that could be implemented at the local governmental level, since we were unable to find an MACC curve for Ciudad Juarez (Table 3). 

Furthermore, we identified mitigation strategies based on scientific knowledge and local indicators (social, political, environmental). The strategies identified were as follows.

- Optimizing routes for public transportation.
- Public lighting system.
- Cargo transportation by rail.
- Urban densification.
- Urban mass transportation.

Step 2: Classification by Mitigation Potential (Environmental Dimension)

Classification of mitigation strategies at the local governmental level provides a roadmap that decision makers can use to align their environmental policies and actions. The strategies for climate change mitigation were classified by their mitigation potential using the MACC curve. The classification is as follows. 

Step 3: Initial Assessment (Technical, Social, Economic, and Environmental Dimensions)

An initial assessment based on scientific knowledge and local indicators (social, economic, environmental) is needed to define the potential of a proposed solution to become policy. A thorough review of the social and political dimensions is critical to achieving policy acceptance; the costs of implementation play a smaller role in decision-making in some cases.

For the purpose of our study, through the bi-weekly public hearings that the local government holds in different areas of the city, we identified some of the most recurrent citizen petitions: (1) improvement and expansion in the coverage of the street lighting system, (2) decrease in the criminal activities and (3) employment generation. From the MACC-derived strategies in Table 3 and the public hearings, we inferred that the street lighting system was promising as a public policy because it could solve the issue raised in the most succesive petition, and also help address the ones raised in the second and third most frequent petitions indirectly through its implementation.

In Table 3 the “optimizing routes for public transportation” strategy has the largest mitigation potential and the most financial viability by its abatement cost; however, when we include the economica, political and social dimensions into the analysis we found that the “energy efficiency of the public lighting system” mitigation strategy was the most viable for local decision makers since it was cost effective and also politically and socially attractive.

Furthermore, we obtained data on the number of lamps, their nominal power, and their application (types of streets: secondary, primary, or principal) from the census performed by the local municipality and on the number of felonies related to the lack of street lighting, and equivalent carbon dioxide emission reductions through energy savings with different public lighting technologies [34,35]. The initial assessment of the data provided three primary outcomes:

1. A census of the Public Services Department showed that there are 100,000 lamps installed in Juarez. Almost 95% of these lamps are used on secondary and primary streets, where the majority of the population lives. Lack of maintenance of the street lighting has resulted in approximately 20,000 lamps (almost one-quarter of the total) being turned off. This has, in turn, led to insecurity, especially in the most impoverished areas of the city.
2. Municipal tax for the right to public lighting (DAP in Spanish) is paid by all citizens and is included in the electric bill. Unfortunately, this tax does not provide enough revenue to meet the costs of electricity for the street lighting, and it is a challenge for the municipal budget to recover the difference.
3. Two types of street lighting are in use in Ciudad Juarez. The first one consists of lighting in upper-middle-class and high welfare areas that has its own infrastructure (poles, lamps, transformers, meters) and is designed based on local street lighting regulations. In these areas, a bill is issued by the national electrical utility to the municipality. For the purpose of our study, we refer to this as the measured street lighting system. The second type of street lighting is primarily found in the poorer and very impoverished parts of the city. This type of lighting lacks a proper infrastructure and is connected directly to the national electric utility grid. This makes it impossible to determine precisely electricity consumption; therefore, the usage is roughly estimated, and the municipalities are billed whether or not the lamps are in use. There are no regulations concerning the design and installation of these lighting systems, and we refer to them as the non-measured or unmeasured street lighting system.

Step 4: Cost-Benefit Analysis (Technical, Economic, and Social Dimensions)

A series of trade-offs among the technical, economic, social, and political dimensions is required to arrive at a policy that would be politically and socially attractive to the city council. For our study, a more efficient model for the street lighting and replacing non-measured street lighting with a measured lighting system was required. 

An initial investment of around 50 to 80 million dollars (depending on the lighting technology) was estimated for carrying out improvements in the street lighting system. We proposed that the debt could be paid through the DAP, energy savings, and federal incentives provided to the municipalities to improve their energy efficiency.

Furthermore, an estimation of health effects in terms of implementing the proposed energy efficiency project was made. The main air pollutants chosen were fine particulate matter (PM_2.5_), sulfur dioxide (SO_2_), nitrogen oxides (NO_x_) and carbon dioxide (CO_2_). The estimation was based on the assumption that electric energy consumption will be reduced by 21,718,147 kWh/year from CFE CT (Federal Commission of Electricity Conventional Thermoelectric power plant) Benito Juarez (Samalayuca I), which is a fuel oil plant, and from CFE CC (Federal Commission of Electricity Combined Cycle power plant) Benito Juarez (Samalayuca II), which is a natural gas plant. Based on this assumption, we used the intake fraction to determine the changes in exposure concentration of PM_2.5_ [36,37]_._

The Harvard Six Cities Study [38], states that an increase of 10 μg/m^3^ in the concentration of PM_2.5_ increases the overall cardiovascular mortality by 9% with a 95% confidence interval of (3%, 16%). The value of statistical life (VSL) for Mexico is $1.2 Million US dollars with a 95% confidence interval of (0.76, 2.9) [39].

The health benefits of improving the energy efficiency of the street lighting in Juarez was estimated to be 11.7 million US dollars/year. The other benefit was the prevention of 9.31 cases/year of mortality by cardiovascular diseases.

Step 5: Institutional Support (Political and Social Dimensions)

There is no standard definition of public policy; this case study defined public policy as a course of governmental action or inaction in response to public problems, reflecting the most important societal values and the conflicts between these values. Policies clarify which of many different values are assigned the highest priority in a given decision [40].

Our strategy was to appeal to some of the more mainstream complex issues, such as the challenge of climate change and improving the quality of life, and that the economic savings be used to support populations in areas that are more at risk. The support of influential non-governmental stakeholders including universities, civil associations, the media, and research centers moved the decision maker from a state of strong influence–low interest to one of strong influence–strong interest.

A multidisciplinary technical committee for the improvement of the public lighting system was implemented. In this committee, the Universidad Autónoma de Ciudad Juarez, Tecnologico de Monterrey Campus Ciudad Juarez, Camara Mexicana de la Industria de la Construccion (a non-governmental organization whose members are professionals in the construction industry) y el Colegio de Ingenieros Mecanicos Electricos y Electricistas del Estado de Chihuahua (a non-governmental group whose members are practitioners in the areas of mechanical, electrical and electronic engineering), and members of the city council participated; the members discussed different options in terms of lighting technology, economic viability, and environmental impact.

The multidisciplinary committee had several meetings to address the different dimensions (technical, economical, social, political, and environmental) involved in the study case to prepare and evaluate different scenarios to be presented to local government officials for the decision-making process.

One of the many trials faced during the study case was that some political actors (mainly from the opposition to the ruling party) were skeptical about the impartiality of some members of the multidisciplinary committee (that were also officials of the local government) on issues such as illumination technology selection, telemetry, and general equipment required to implement the project. To include different interests from the public and private sector stakeholders, the multidisciplinary committee produced different public policy scenarios for the decision-making process, which included the preferred options from various actors.

Once the city council selected one of the scenarios that were presented, some political actors that were members of the multidisciplinary committee and had signed the scenarios that were produced, denounced the policy-making process since the selected scenario was not their preferred choice. 

A spatial analysis was carried out on the collected information to produce thematic maps that could illustrate the relationship between street lighting and socio-demographic indicators, with two primary objectives: (1) obtain a broad understanding of the problem that requires solving and of the potential for climate mitigation, and (2) develop a simple, graphical format for communicating the problem, potential, and local indicators to decision makers for the support stage [41,42,43,44,45,46,47,48,49,50,51,52]. 

Other thematic maps obtained during this initial assessment indicated crimes or felonies related to the lack of street lighting. These maps included robberies, murders, vehicle accidents, and pedestrian accidents, and helped to highlight the urgent need to address security and crime on the political agenda [53].

After a thorough review of several street lights manufacturers, we proposed to the local government that LED lamps would provide the highest energy savings (~64%) relative to the existing lights, have a strong positive social effect in terms of improved security, and help avoid emissions of more than 30,000 tCO_2_e per year as a result of energy savings.

Step 6: Policy Proposal (Technical, Social, and Economic Dimensions)

In this step, a very close working relationship is developed with the local government to decide what needs to be added or updated in the case of existing environmental policies or the creation of new ones, supported by the technical, economic, and socio-political aspects of the scientific report.

We proposed that the existing street lighting regulation is updated (after almost ten years with no changes) to include energy efficiency and climate change mitigation as design key variables. Our proposal recommended the use of best available technology for street lighting and prohibition of sodium vapor lamps in future developments. This update was suggested to ensure continuity of this effort regardless of any political changes in the local government. Through the efforts of the multidisciplinary technical committee, five different scenarios were presented for decision-making and their possible implementation through policy proposal.

Step 7: Decision Making (Political and Social Dimensions)

A close relationship and dialogue with the local government is required in this step as well to explain the policy proposal in detail to different political stakeholders (Mayor and City Council). Final modifications at this stage could be made to support the decision-making.

Based on the results of Steps 1–7, we proposed a public policy, which consisted of updating the street lighting regulations, more than a decade after their first publication, and addressing climate change mitigation and energy efficiency as key points in the current and future public lighting designs. The policy was approved unanimously by the city council and has already been enforced. Several branches of the local government, with the authors of this paper as advisors, were commissioned with the task of creating an implementation plan for the public policy.

Step 8: Policy Implementation (Political, Technical, and Economic Dimensions)

As we prepare this content (March 2015), the local government is in the process of obtaining the financial resources, *i.e.*, 50 million dollars, to change the existing 100,000 lamps, replace the non-measured street lighting system with a measured one and expanding the coverage of this service to the entire population. 

The implementation of this policy is dependent on the decision makers at the local government level; however, by completing Step 7 and through maintaining non-governmental institutional support, we believe it is only a matter of time implemented the policy. Local government officials specifically requested that the group working in an advising role in this study case do not participate in the matters related to the funding of the project since there were some political issues on this subject and they wanted to have control of this area.

Through the implementation of our methodology, we were able to improve the energy efficiency of street lighting, through the use of light-emitting diode (LED) lamps, with energy savings of 64% and emission reductions of ~30,000 tCO_2_e/year. These savings achieved by changing the 100,000 existing streetlights. Following our successful experiment, the street lighting regulation was updated to ensure that energy efficiency and climate change mitigation taken as fundamental considerations in the current and future design and applications for street lighting.

## 4. Discussion 

The implementation of the proposed multidimensional methodology for building climate change mitigation policies in urban areas in Mexico has presented and explained with the help of a case study of energy-efficient street lighting in Juarez, Chihuahua, Mexico. 

We had a leadership-absent local government scenario which poses an opportunity for the members of the research project to take a leadership role derived in communication with decision makers, strengthen the institutional structures available to implement the recommendations from our research encouraging decision and action. The stakeholders involved in this process at the local level in Ciudad Juarez are very similar in their views towards science and government as shown in other studies, allowing us to present a methodology which in principle can apply to other geographical areas in Mexico or internationally. 

While multidimensional transdisciplinary research is considered as an important aspect towards sustainability, the concept is complex and still under development and empirical experimentation. Some potential obstacles found during the study case which originates from the fact that scientist from different disciplines and academic traditions need to collaborate and integrate their knowledge and experience [54]. Also, an effective integration of participants from society, often with different conflicts interest, is another obstacle to be overcome more efficiently by empirical research [55].

## 5. Conclusions

One of the challenges for climate change mitigation policies arises from the interactions among the technical, environmental, economic, social, and political dimensions that increase the complexities for the decision makers, including timeframe mismatches between researchers and election cycles. Our approach to convert mitigation strategies into public policies is an attempt to create a bridge between the scientific community and the decision makers at the local governmental level (urban areas) by providing reliable information to support the decision-making process.

A new protocol for communication between the decision-makers and the scientific community is essential to achieving the common goal of implementing climate change mitigation policies. We learned from this experience that public health impacts and spatial analyzes are powerful tools to communicate complex technical, economic, environmental, and social information to decision makers in a clear manner and facilitate the decision-making process. Further work using the proposed methodology regarding of other mitigation strategies and a further refinement of the steps are needed. 

## Figures and Tables

**Table 1:** Characteristics of science and government.

Science	Government
Probability accepted	Certainty desired
Problem-oriented	Service oriented
Risk accepted	Risk intolerable
Anticipatory	Time at the next election ends

**Table 2:** Description of the eight steps of the methodology and the dimensional interactions involved in each step.

Steps	Description	Dimensions
1. Identification of mitigation strategies	Identify mitigation strategies for climate change at the local governmental level.	Social, political, and environmental
2. Classification by mitigation potentials	Classify mitigation strategies according to their CO_2_ impacts.	Environmental
3. Initial Assessment	Perform initial assessment using scientific knowledge and local indicators	Technical, social, economic, and environmental
4. Cost-benefit analysis	Analyze costs, benefits, and trade-offs to provide an attractive policy proposal regarding each dimension	Technical, economic, and social
5. Institutional support	Access governmental and non-governmental support	Political and social
6. Policy proposal	Propose and select a proper communication protocol for decision makers	Technical, economic, and social
7. Decision making	Approve policy if benefits are greater than costs	Political and social
8. Policy implementation	Enact policy	Political, economic and technical

**Table 3:** Classification of strategies by mitigation potential through Mexico MACC curve for the period 2009–2030.

Mitigation Strategies	Mitigation Potential	Abatement Cost
Optimizing routes for public transportation	350 MtCO_2_e	−95 USD/tCO_2_e
Energy efficiency of the public lighting system	100 MtCO_2_e	−25 USD/tCO_2_e
Cargo transportation by rail	200 MtCO_2_e	−90 USD/tCO_2_e
Electricity generation by methane gas in city dump	300 MtCO_2_e	−95 USD/tCO_2_e
Urban mass transportation	150 MtCO_2_e	−50 USD/tCO_2_e
